# Mapping the space of dementia with EEG in stained glass

**DOI:** 10.3389/frdem.2026.1820301

**Published:** 2026-06-25

**Authors:** Mick O’Kelly, Serggio C. Lanata, Jonathan K. Kleen

**Affiliations:** 1 Global Brain Health Institute, University of California San Francisco, San Francisco, CA, United States; 2 Memory and Aging Center, University of California San Francisco, San Francisco, CA, United States; 3 Department of Neurology, University of California San Francisco, San Francisco, CA, United States; 4 Weill Institute for Neurosciences, University of California San Francisco, San Francisco, CA, United States

**Keywords:** electroencephalography, light, quality-of-life, signal processing, spectrogram

## Abstract

Dementia conditions, including Alzheimer’s disease, are progressive disorders for which effective treatments remain under development. Accordingly, interventions that improve quality of life are an important focus of patients, caregivers, and providers, including traditional art forms like painting and sculpture. In this Perspective article, we discuss the use light as a unique form of art to create transformative spaces that stimulate the senses and potentially support wellbeing. We outline an art-science collaborative initiative in which the patients themselves contribute, including through the recording and conversion their EEG brainwave activity to 2D time-frequency spectrogram representations. These representations are transferred to the ancient art of stained glass and installed into the patient’s own home. This intervention creates a novel dynamic environment of illumination and colour saturation, derived from the patient and in sync with ever-changing solar and climate influences. Details of the approach and options for meaningful patient-centred customization are described, with illustrations of the procedures and resulting installations, along with thoughts for future improvements.

## Introduction

Alzheimer’s disease (AD) and other dementias are slowly progressive neurodegenerative disorders. The accelerating incidence and prevalence of AD and other dementias is a major societal issue and will drive trillions of dollars in costs to society globally over the coming decades (Chen et al., 2024; Li et al., 2022). Due to a dearth of effective pharmacological treatments, methods to improve behavioural and cognitive engagement are a major cornerstone of dementia care.

A central theme in the course of dementia is loss: loss of time, communication, personality, meaningful interactions, and other dynamic aspects. In tandem, the lives of caregivers and other family and friends are profoundly affected as well. “Dementia is a thief” wrote the wife of one of our patients diagnosed with AD. *“Dementia has stolen our sweet together memories, our ability to plan for the future, and even our mundane day-to-day communication. Feelings are easily hurt. The ‘soul-mate’ part of marriage evaporates and is replaced with the ‘business partnership’ of navigating the slow but ever-increasing losses and changes.”*

Much of patient-doctor relationship in neurodegenerative conditions similarly centres on what is lost: functions, memories, and the quality of interactions. Clinicians use formalized cognitive evaluations to measure memory loss and the devastating impact it has on quality of life (QOL), including one’s identity, and sense of self. These latter aspects are, after all, some of the most salient and sacred parts of being human.

It is challenging to refocus this narrative, and our minds, to the reality that the brilliance of life continues during the journey of illness. Focus is rarely given to what positive phenomena one can see, feel, or otherwise experience as dementia progresses. In other words, it can be understandably easy to miss novel perspectives and experiences that are gained, because of the difficult, chronic, and progressive context of illness. Could novel stimulating contexts and environments break that mold and lead to new and positively changing experiences?

### Art in dementia care

An established approach to addressing QOL during progressive neurodegenerative disease is through artistic activities (Pongan et al., 2017; Wyatt and Boddington, 2025; Ward et al., 2021; Jeppson et al., 2022). Prior studies on painting, drawing, and other art forms to improve cognitive stimulation have shown improved constructive engagement, communication, and/or QOL among patients with various forms of dementia (Jeppson et al., 2022; Hattori et al., 2011). Importantly, art endeavours can also improve the well-being of caregivers of patients with dementia (Leung et al., 2022).

How might the artistic design of environments (spaces) be integrated as a tool in clinical care for wellbeing? Perhaps if driven, embraced, and guided by virtue of the person’s own experiential neural activity during their disease progression. Art works within the space of cognition, after all. A stimulating environment influences the brain dynamically, and the brain in turn can influence the environment. Combining art with science and medicine produces new opportunities to create transformative, positive spaces for individuals living with dementia. This collaborative mindframe could plausibly help shift the narrative and emphasis from loss to the active and creative connections in the brain and mind.

### Light as art

In art institutions, light is used to guide and focus our attention, with gallery spotlights as an example. But the experience of light is not only a retinal encounter, it is an immersive and embodied experience, saturating the senses, affecting one’s circadian rhythm, sleep patterns, and body metabolism. There is evidence to indicate that exposure to cool light of moderate intensity diminishes agitation and anxiety, creating a place of calm (Figueiro et al., 2014; Goudriaan et al., 2021; Wahnschaffe et al., 2017). It may be more energizing when an art-neuroscience collaboration can take us on a different parallel journey, imagining new identities and forming new memories while being in the immersive moments of light and colour.

Light not only reveals, but is a revelation in itself. In other words, to see and experience light and colour can be understood through its own specific reflective view of the senses. One becomes aware of being present in its immersive quality. There is no obligation to project meaning onto this experience, just to be present in the moment is a reward. To quicken the heart, frees up ones thought.

We sought an approach that redirects focus on loss to the overflow of thinking that dynamic and ever-changing light could enable. To capture this overflow, our initiative, which we term Mapping the Space of Dementia, aimed to capture brain activity as a moment in time, transformed to colour and mapped onto the ancient art of stained glass to be installed in the participant’s home environment. By making the brain activity visible, the participant observes their thoughts return as a medium of light through this stained glass window, where the cosmic rays and changing weather dynamically illuminate the milieu in saturated light and colour.

**Figure 1:**
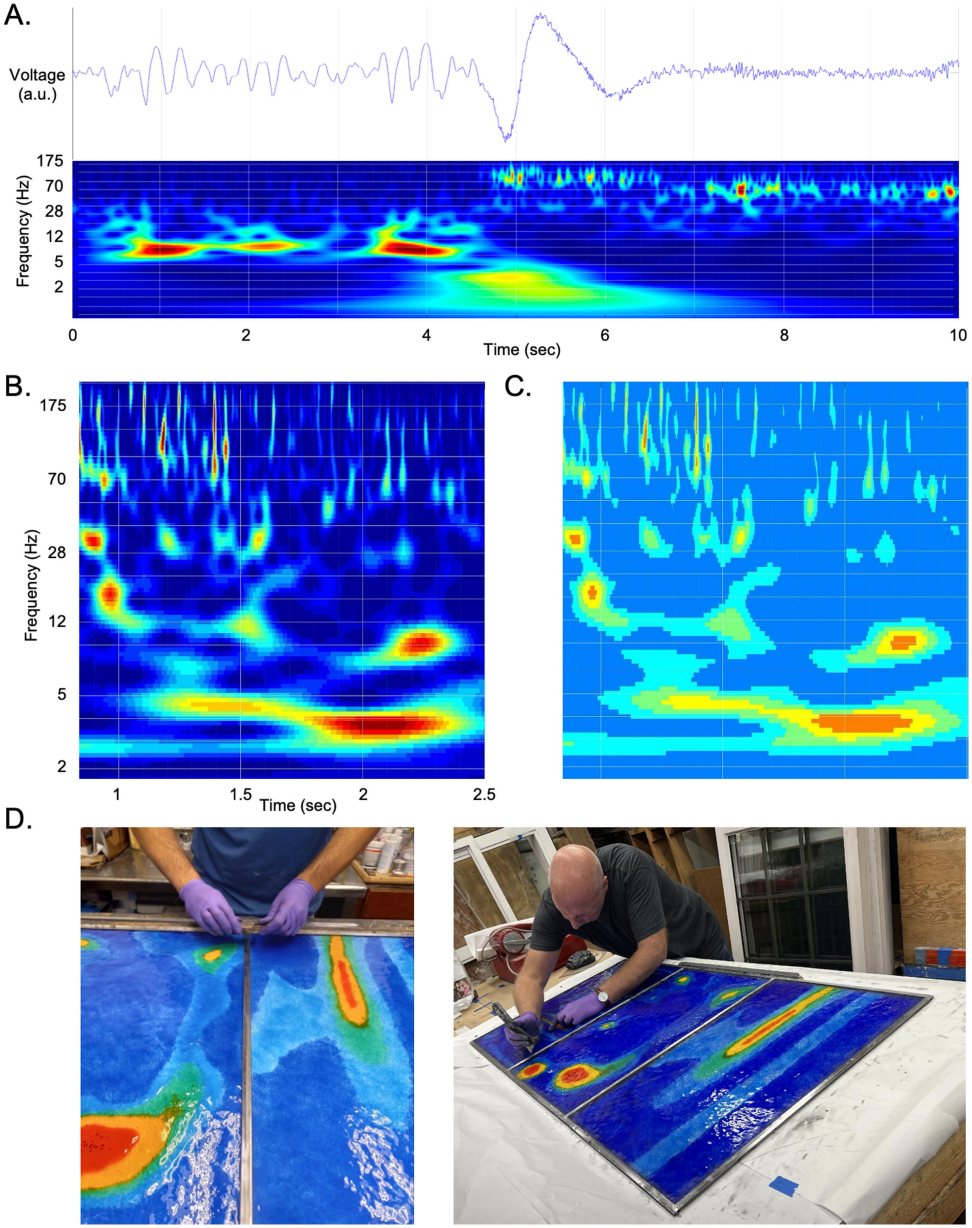
EEG spectrogram in stained glass. **(A)** Example of a recorded EEG voltage signal (top panel) demonstrating how frequency admixtures (superimposed rhythms) embedded in the signal change the spectrogram colour representation accordingly over time (lower panel). **(B)** The unique spectrogram pattern from a hand-selected EEG signal segment from a patient, comprised of a Morlet wavelet transform using an O1-O2 bipolar EEG signal and plotted using a “jet” colourmap with 15 levels (steps) of colour. **(C)** Same spectrogram as A simplified to 5 colour steps. **(D)** Stained glass manufacturing process with the artist working on the spectrogram from **B**.

A natural candidate to capture spontaneous brain activity is electroencephalography (EEG). Intricate brainwave patterns can be transformed through neural signal processing and coloured in comprehensive detail using a spectrogram, a 2-dimensional representation in colour that represents the dynamic strength of each frequency at each moment in time. Raw EEG waveform traces can in fact be reconstructed from the pattern represented in the spectrogram, a two-way relation fitting the bidirectional relation of the patient and the dynamic light environment (Figure 1A).

Three layers of dynamic experience are blended into this immersive environment: (1) the spontaneous brain activity patterns originally captured from the patient’s recording (though its spectrogram form then becomes fixed in the artwork), (2) the activities and interactions of the patient in the space of the room into which light from the stained glass window is projected, and (3) the dynamic changes of the projected light in the room as the sun moves throughout the day along with varying clouds and other weather patterns. The self-produced beauty of light, colour, and human experience thus becomes ever-changing in this space throughout the patient’s journey.

Below we detail the methodology of the data acquisition/conversion process for rendering EEG data into spectrograms suited for stained glass installations. We also describe the potential versatility of many steps that can be fit according to patient preference. This underscores that the art-neuroscience artwork is not made *for* the patient, but *with* them, through their own EEG signals, preferences in colour, chosen window, and the specific lived moment chosen from the recording.

### Approach

Below we include detail to understand and replicate our approach, accordingly with guidance on overcoming technical challenges we encountered in our initial series of patients. We worked with the initial patient at UCSF Medical Center in San Francisco, CA, USA (*n* = 1) and four subsequent patients at St. James Hospital in Dublin, Ireland (*n* = 4). All had a neurodegenerative disorder diagnosis and expressed interest in this art & neuroscience initiative. This art and neuroscience initiative was deemed as not requiring ethical approval by both the Office of Research & Innovation at St. James Hospital and UCSF Medical Center. Video-EEG was obtained with electrodes placed in standard 10–20 montage with EEG recorded at 512 Hz (both sites utilized recording equipment and software from Natus Medical Inc., Middleton, WI, USA). The recorded data was exported as a de-identified EDF file and imported into MATLAB (The Mathworks Inc., Natick, MA, USA). No filtering was applied aside from notch-filtering to remove 60 Hz line noise artifact (50 Hz for the Dublin recordings) and harmonics.

A personally meaningful moment was selected for each recording. For the first patient this was a brief moment on video in which the patient and their spouse were looking and smiling at each other. The other four patients were asked prospectively what they preferred to focus on during the recording. One chose to look through family photo albums, and the rest chose to listen to a particular favourite piece of music. EEGs had been annotated prior to exportation by time-marking a segment of 2 to 4 s during the activity, avoiding sections with electrical or movement artifact.

#### Electrode choice

The EEG contains brainwave signals from many electrodes, and so a relevant bipolar pair of electrodes was chosen for rendering each patient’s spectrogram. Bipolar pairs can help reduce artifact compared to referential recordings and tend to convey a rich frequency admixture rendering intricate spectrogram patterns (Caldwell et al., 2025). For the two cases in which the focus was a visual context (social interaction, looking at photos), a bipolar pair between the O1 and O2 electrodes was utilized to capture signals largely reflecting visual cortical activity. Two patients listened to a favourite classical music piece (no lyrics) for which neural activity from the right temporal region was used (T4, referenced to C4 for clean signal) reflecting auditory neural processing related more toward musical tones and melody. One patient listened to a favourite song including lyrics for which neural activity from the left temporal region was used (T3, referenced to C3 for clean signal), reflecting neural processing related more toward auditory and language components of music.

#### Signal processing

An EEG segment of EEG of 2 to 4 s was chosen according to annotations created during video-EEG review. A power spectrogram was created by converting the time-domain voltage-based signal into the frequency domain (Figure 1A). We utilized a wavelet transformation given the balance of high temporal and frequency resolution, though other transforms (e.g., sliding-window fast Fourier transform, Hilbert transform) are feasible as well.

Neural data follows a power law in which lower frequencies are naturally higher in amplitude, which can dwarf and thus obscure the display of higher frequencies which are progressively lower in relative amplitude. This makes it difficult to interpret high and low frequencies simultaneously within the same spectrogram, but scaling by frequency can help relative balance. We performed a normalization in which the amplitude of each frequency was multiplied by the frequency number itself, scaled to an exponent, and the square root was then taken across all frequencies to adjust for amplitude skew before rendering the spectrogram.

The palette of colours using step-wise colour values and their sequential order (“colourmap”) represented the signal magnitude (power) at each timepoint and frequency (Figures 1A–C). To improve the personalization and aesthetics of the spectrogram, patients chose their preferred colourmap. The number of steps of colour is specified, similar to a topographical land map (e.g., steps in elevation). More colour steps add spectrotemporal detail yet increase manufacturing complexity of the stained glass rendering process by increasing numbers of colours and step contours (Figures 1B,C). We typically chose 10 to 15 levels for colourmaps to balance trade-offs between spectrotemporal pattern complexity, colour aesthetics, and manufacturing feasibility.

#### Stained glass manufacturing process

This colourmap of spectral components was translated onto the participant’s window using the ancient art of stained glass in collaboration with master stained glass experts and their workshops (Lenox Stained Glass in San Francisco, CA and Glasshammer Studio in County Offaly, Ireland). Height and width measurements of the preferred window in the patient’s residence were taken for a base frame. A large printout of the final spectrogram was created on white paper, and contours of spectrogram magnitude steps were noted according to colour.

**Figure 2:**
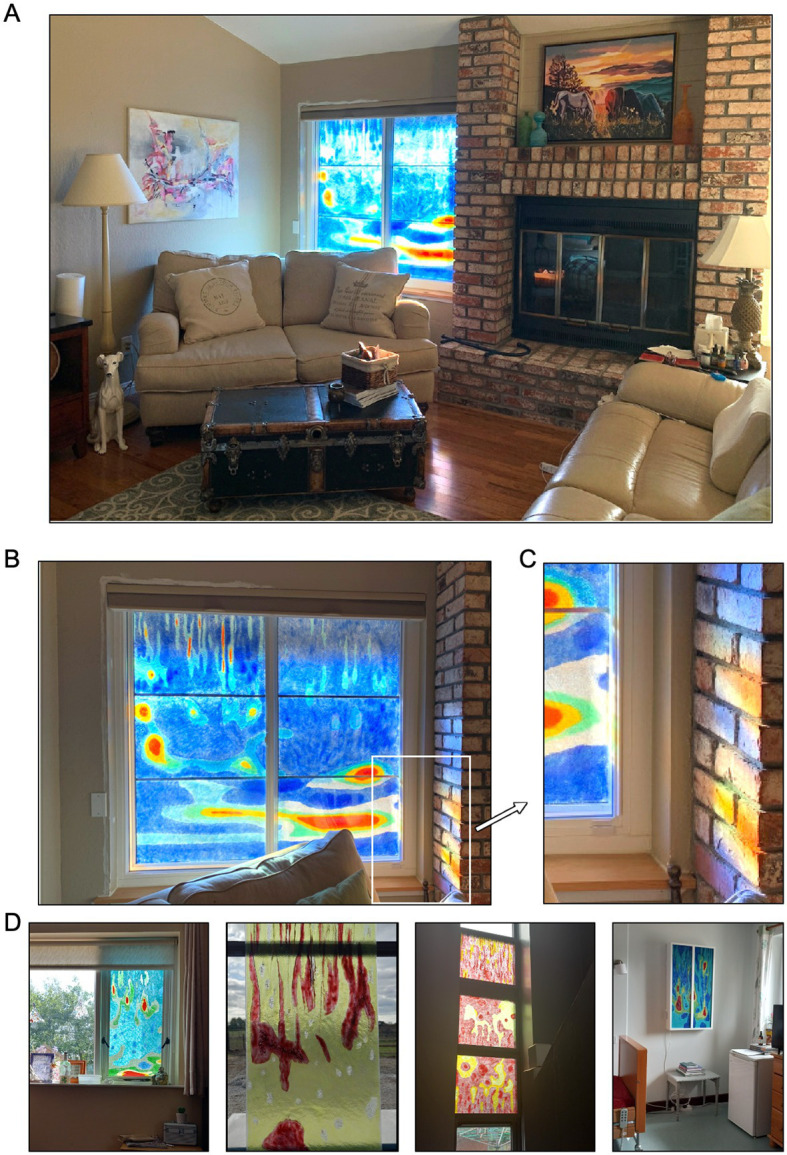
Home installation and dynamic lighting effects. **(A)** Installation of stained glass spectrogram from the EEG spectrogram from Figures 1B–D into the patient’s home living room. **(B)** Demonstration of sunlight filtered through the stained glass, influencing the internal environment and dynamic state of the home space. **(C)** Closer view of interior lighting detail cast by the stained glass piece. **(D)** Additional installations of stained glass spectrograms (participants 2–5), including lightbox version (right-most panel).

Traditional methods of coloured glass production were performed (Figure 1D). In brief, clear float glass was placed on top of the colour spectrogram and glass frit (crushed glass in crystal or powder form) was manually placed, carefully following the colour pattern. The glass was fired in a kiln several times to acquire translucency and desired colour density. We used lead came strips soldered together to bond glass panels and assembled them to fit the unique window frame. Dimensions varied and were bespoke to each environment. Upon completion, each stained glass artwork was delivered to the corresponding patient’s home and installed in the dedicated window frame by the artist (M.O.; Figure 2).

### Patient-centred art-neuroscience collaboration

It is important to emphasize that we propose the light-based stained glass artwork itself, and its creative process incorporating the patient’s contributions, as a plausible intervention in dementia care (i.e., not specific light frequencies or other objective scientific features of the art). We suspect this approach may plausibly convey benefit similar to other art-based approaches though further objective study would be needed to support this claim. Crucially, the personalization of colour and design, their aesthetics, and symbolism to the patient (e.g., their chosen meaningful lived moment during the recording) in fact represent crucial contributions by the patient in the creation of the artwork as a collaboration between the patient, the artist, and the clinical provider(s)/team.

Since the patient is integral to co-authorship of the art-science collaboration, the physical installation represents a significant moment in which the owner of the brain circuitry sees their thoughts return through stained glass. As the sun changes its angle, as the seasons change, and as the weather oscillates between sunny and overcast days, a constantly changing solar experience is created (Figures 2B,C). One patient lived in a residential home in which altering windows was not feasible, and so a light box was created for the artwork. Otherwise, window locations were chosen by each patient with guidance by the artist, with the goal of optimizing the available use of light, ideally where light entry angles from solar positioning could change illumination and colour patterns in the room environment throughout the course of each day (Figure 2D).

## Conclusions and future directions

This ongoing creative work brings together scientists, clinicians, care workers, caring partners, people living with dementia, and an artist. The ambition for the pilot collaborative work between art and neuroscience was firstly to develop a project concept that could be delivered with feasible outcomes. Secondly it was to develop hybrid knowledge that had the capacity to start with individual participants and scale up to healthcare provision institutions. A parallel goal is to build and develop a collaborative art and neuroscience initiative that produces new hybrid knowledge, in which the assemblage is greater than the sum of its unique parts. In other words, here we describe different modes of thinking and tropes of representation to frame a position for creative interventions for people living with AD and other dementias.

Could interventions combining novel light art and neuroscience be a creative tool in innovative approaches in dementia services, going beyond traditional care and support frameworks? As opposed to work utilizing specific intensity, spectrum, and timing features of light interventions, the artwork here is a more patient-specific (heterogenous) intervention to produce transformative spaces using light as a dynamic medium. However, the positive effects of creating transformative spaces that stimulate the senses for persons living with AD have been clinically demonstrated (Day et al., 2000; Halsall et al., 2023). Thus the significance of producing space as a mitigating factor in supporting cognitive reserve should not be underestimated. Nevertheless, while initial feedback from the patients in this cohort was positive (e.g., enjoyable, meaningful), more objective evaluation of the utility of specific features of EEG stained glass interventions (e.g., degree of patient contribution, specific colours/colourmaps, tailoring complexity/clarity of the design to avoid unintended cognitive load) should be the focus of future study. In addition, potential costs of stained glass manufacturing and professional installation, along with sparse availability of professional stained glass studios, are potential limitations of the approach described herein.

Space and light affect our state of mind and the way we organize the complexity of contemporary living. This is experienced within constructed environments, for example classrooms, factories, galleries and museums, hospitals, care home, and the domestic sitting room. These are places of social encounters, sounding out different levels of engagement and belonging in which one can change the context, creating new memories—a narrative for living. These settings, as well as art galleries and other public artwork exhibitions, or even public spaces, could be thought of as avenues for scaling light-based artwork.

Installations of EEG stained glass artwork in a medical environment could move the work from a unique to a collective encounter. There is a perception that we live in our unique memory. Alternatively, we live in a collective memory of other people’s images and stories, perhaps an impossible memory in the way we always recall memories that are never quite the same as the memories lived. To live outside of time and language is to live in intuition, where remembering is not the opposite of forgetting. Mosaics of colour activated by the weather could influence the collective memories of patients and staff, with an ambition to provoke a feeling of presence shrouded in light. The encounter may shift between an immersive outer body experience or grounded where one is conscious of gravity and perspective. This could plausibly be an energising experience for all who live and/or work in that space.

Art installations and exhibitions are also forms of publication and distribution of knowledge. They advance an awareness of the creative imagination, brain health, and a space that nurtures wellbeing. Space is something we produce, and spatial design can embody an integrated tool for clinical care environments for people living with dementia. Future installations in a hospital or clinical waiting room, or long-term care facility, would enable the ever-changing experience to be available to staff, patients, and visitors, all influenced by the unique brain wave patterns of an individual who was in turn influenced by that same environment.

This work advances ideas for art and science to liaise with architectural practices, providing tailored design strategies for people who are cognitively and spatially challenged. Equipping spaces with light, physical, material, linguistic, conceptual, perceptual, and ephemeral influences enables new experiences, immersed in moments of light and colour. Despite the focus on loss being predominant in dementia progression, we speculate that artistic transformations like the approach herein may provide perpetually meaningful, and even gainful, experiences.

## Data Availability

The raw data supporting the conclusions of this article will be made available by the authors, without undue reservation.
